# Histone deacetylase HDAC4 participates in the pathological process of myocardial ischemia-reperfusion injury via MEKK1/JNK pathway by binding to miR-206

**DOI:** 10.1038/s41420-021-00601-1

**Published:** 2021-09-15

**Authors:** Qingman Li, Lijie Zhu, Fangqing Niu, Qingmin Li, Che Wang, Honghui Yang, Chuanyu Gao

**Affiliations:** 1 Department of Cardiology, Henan Provincial People’s Hospital, Zhengzhou University People’s Hospital, Zhengzhou, 450003 P. R. China; 2 Department of Cardiology, Central China Fuwai Hospital, Central China Fuwai Hospital of Zhengzhou University, Zhengzhou, 451464 P. R. China

**Keywords:** Apoptosis, Cardiac hypertrophy

## Abstract

Histone deacetylases (HDACs) and microRNAs (miRs) have been reported to exert pivotal roles on the pathogenesis of myocardial ischemia-reperfusion injury (MIRI). Therefore, the present study was performed to define the underlying role of HDAC4 and miR-206 in the pathological process of MIRI. An IRI rat model was established. The interaction between HDAC4 and the promoter region of miR-206 was determined using ChIP, and that between miR-206 and mitogen-activated protein kinase kinase kinase 1 (MEKK1) was determined using dual luciferase reporter gene assay. After the loss- or gain-of-function assay in cardiomyocytes, western blot analysis, RT-qPCR, TUNEL, and ELISA assay were performed to define the roles of HDAC4, miR-206, and MEKK1. Up-regulation of HDAC4 and down-regulation of miR-206 occurred in rat myocardial tissues and cardiomyocytes in MIRI. HDAC4 down-regulation or miR-206 up-regulation contributed to reduced cell apoptosis and the levels of tumor necrosis factor-alpha (TNF-α), interleukin-6 (IL-6), and malondialdehyde (MDA), while elevating the superoxide dismutase (SOD) and glutathione (GSH) contents. Meanwhile, HDAC4 silencing promoted the expression of miR-206, which targeted and negatively regulated MEKK1. Then inhibition of JNK phosphorylation reduced the cardiomyocyte apoptosis to alleviate MIRI. Coherently, HDAC4 silencing could up-regulate the expression of miR-206 to reduce cardiomyocyte apoptosis and inhibit oxidative stress, and exerting a protective effect on MIRI via the MEKK1/JNK pathway.

## Introduction

Myocardial ischemia-reperfusion injury (MIRI), a manifestation of cardiomyocyte apoptosis induced by ischemia-reperfusion (IR), is a crucial cause for myocardial damage and subsequent heart failure, resulting in high morbidity and mortality worldwide [1]. The innate immune system and following inflammatory responses possess vital significance during the process of myocardial damage extension [2]. Currently, the clinically adopted management protocols for MIRI include ischemic pre-conditioning, pharmacological intervention, and physical interventions like hypothermia or electrical stimulation [3]. However, the advent of MIRI is inevitable due to limitation of reperfusion as the only established therapeutic modality for acute myocardial infarction till date, which raises concern for the development of other effective strategies to reduce IRI [4]. Hence, the current study was devised to explore a novel therapeutic target to alleviate MIRI at a molecular level.

Histone deacetylases (HDACs) serve as critical modulators for mediating myocardial protection and the survival of cardiomyocytes [5]. More notably, the inhibition of histone deacetylase 4 (HDAC4) has been demonstrated to confer significant cardioprotective effects against hypoxic injury [6]. Also, HDAC4 down-regulation functions as a critical stimulant for myocardial repair [7].On the other hand, a study has demonstrated that HDAC4 is a target of microRNA-206 (miR-206) in the regulation of myogenic differentiation [8]. Moreover, the specific genetic silencing of HDAC4 leads to up-regulation of miR-206 in rhabdomyosarcoma, which is an aggressive soft-tissue cancer characterized by disturbed myogenic differentiation [9], indicating that miR-206 is specifically regulated by HDAC4. Meanwhile, HDAC4 accumulation prevents the hypertrophy of myogenic cells triggered by miR-206 inhibition [10]. Inherently, miRNAs are defined as a large group of post-transcriptional regulators with approximately 30% of human protein-coding genes [11], which essentially function in IRI by altering crucial elements of multiple pathways detrimental to the fate of IRI [12–14]. Furthermore, miR-206 overexpression has also been shown to possess the ability to attenuate MIRI in rats [15]. In addition, miR-206 is implicated in the regulation of skeletal muscle differentiation via suppression of multiple factors of the c-Jun N-terminal kinase (JNK)/mitogen-activated kinase-like protein (MAPK) pathway such as mitogen-activated protein kinase kinase kinase 1 (MEKK1) and MAP kinase kinase 7 [16], which is highly suggestive of a novel regulatory mechanism involving HDAC4, miR-206, and the MEKK1/JNK pathway. Meanwhile, MEKK1 is a protein kinase activated by mitogen and has been demonstrated to be implicated in cardiac remodeling [17]. JNK is a protein kinase that can be activated by stress or mitogen, and incorporation of the mitochondrial JNK pathway has been indicated in ischemic myocardial dysfunction [18]. Of note, JNK activation has also been implicated in cardiac IRI by previous studies [19]. Currently available JNK inhibitors hold great therapeutic potential for the treatment of cerebral and MIRI considering their cardioprotective and neuroprotective properties [20]. Meanwhile, the suppression of MEKK1 and JNK can also confer protective effects against cardiac hypertrophy and heart failure [21]. Conjointly, the existing evidence suggests that repression of HDAC4 can exert crucial cardiac protective effects in the regulation of miR-206-mediated MEKK1/JNK pathway during the process of MIRI. Consequently, the current study was conducted to confirm the aforementioned hypothesis and define the underlying molecular mechanisms of the HDAC4/miR-206/MEKK1/JNK axis in the pathological process of MIRI.

## Results

### HDAC4 was up-regulated in cardiomyocytes after MIRI and HDAC4 silencing could alleviate myocardial injury in vivo

**Fig. 1:**
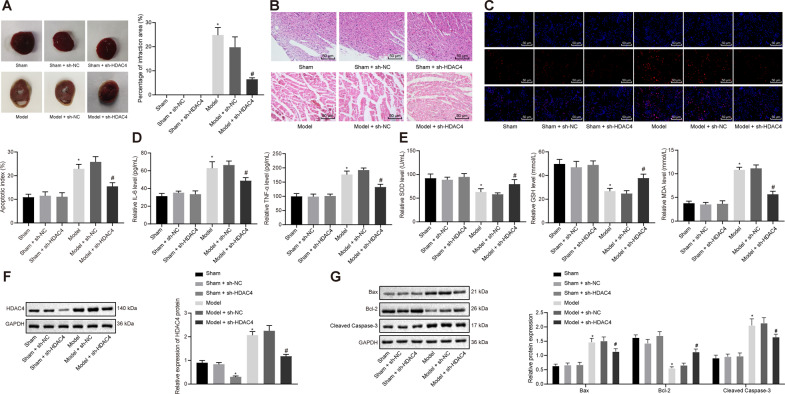
HDAC4 is up-regulated in rat myocardial tissues after MIRI and its silencing alleviates myocardial injury in vivo. **A** Analysis of the myocardial injury area of rats by TTC staining (*n* = 5 for rat in each group). **B** Representative images of the myocardial injuries evaluated by H&E staining (*n* = 10; scale bar = 50 μm). **C** Apoptosis of cardiomyocytes detected by TUNEL assay (*n* = 10; scale bar = 50 μm). **D** Serum levels of IL-6 and TNF-α in peripheral blood of rats measured by ELISA (*n* = 10). **E** Determination of SOD, GSH, and MDA contents in rat myocardial tissues (*n* = 10). **F** Protein expression of HDAC4 in rat myocardial tissues detected by western blot analysis, normalized to GAPDH (*n* = 10). **G** Protein expression of Bax, Bcl-2, and cleaved Caspase-3 in rat myocardial tissues detected by western blot analysis, normalized to GAPDH (*n* = 10). ^*^p < 0.05 vs. the sham-operated rats; ^#^*p* < 0.05 vs. the rats with MIRI. Data were all measurement data and expressed as mean ± standard derivation. Comparisons among multiple groups were analyzed by one-way ANOVA, followed by Tukey’s post hoc test.

To explore the role of HDAC4 in IRI, we first established an IRI rat model. Analysis of the myocardial tissues with 2,3,5-triphenyltetrazoliumchloride (TTC) staining (Fig. 1A) demonstrated that the presence of an infarct area in the area at risk (AAR) was much higher in the IRI rats than that in the sham-operated rats, while the percentage of infarct area in AAR was found to be reduced in short hairpin RNA (sh)-HDAC4-treated IRI rats compared to IRI rats. In addition, hematoxylin-eosin (H&E) staining illustrated that the sham-operated rats presented with neatly and compactly arranged cardiomyocytes, evenly stained cytoplasm, same nucleus size, and neat myocardial fibers without obvious fracture. However, the cardiomyocytes were disorderly arranged with hypertrophy, myocardial fibers showed rupture and dissolution, and interstitial collagen accumulation was accompanied by myocardial fibrosis and necrosis in IRI rats, which indicated that myocardial ischemia induced these myocardial morphological changes. Obvious improvements were noted in myocardial injury in IRI rats treated with sh-HDAC4 (Fig. 1B).

The results of terminal deoxynucleotidyl transferase-mediated dUTP nick-end labeling (TUNEL) assay ascertained that compared with the sham-operated rats, a high proportion of apoptotic cardiomyocytes was observed in IRI rats, while silencing HDAC4 led to reduced cell apoptosis in IRI rats (Fig. 1C). It has been well documented that cytokines, including tumor necrosis factor-alpha (TNF-α) and interleukin-6 (IL-6), are associated with the pathophysiology of cellular dysfunction in IRI [22]. As a result, enzyme-linked immunosorbent assay (ELISA) was conducted to investigate the levels of IL-6 and TNF-α in the serum samples of rats from each group. The results revealed significantly elevated serum levels of IL-6 and TNF-α in IRI rats, while further HDAC4 silencing notably reduced the serum levels of those proinflammatory cytokines in IRI rats (Fig. 1D). Also, research has suggested an association between oxidative stress subsequent to IRI and cardiomyocyte death during the IRI [23].

In order to investigate whether HDAC4 silencing affects oxidative stress, we evaluated the expression levels of antioxidant enzymes and the content of malondialdehyde (MDA). It was found that the contents of superoxide dismutase (SOD) and glutathione (GSH) were drastically decreased but MDA content was increased in the IRI rats compared with the sham-operated rats. Additionally, HDAC4 silencing reversed the findings in rats with IRI (*p* < 0.05) (Fig. 1E). The results of western blot analysis revealed down-regulated expression of HDAC4 in sham-operated rats treated with sh-HDAC4 as compared to sham-operated rats and sham-operated rats treated with sh-negative control (NC). The expression of HDAC4 was up-regulated in IRI rats relative to sham-operated rats (*p* < 0.05). HDAC4 was reduced in IRI rats treated with sh-HDAC4 (*p* < 0.05; Fig. 1F). Moreover, up-regulated pro-apoptotic factors (B-cell lymphoma-2-associated protein X (Bax) and cleaved Caspase-3) expression and down-regulated anti-apoptotic factor B-cell lymphoma-2 (Bcl-2) expression were witnessed in rats with IRI. HDAC4 silencing reduced Bax and cleaved Caspase-3 expression and elevated Bcl-2 expression in the IRI rats (*p* < 0.05; Fig. 1G). The aforementioned data supported that HDAC4 was robustly induced in IRI and its silencing essentially mitigated myocardial injury.

### HDAC4 silencing alleviates injury of cardiomyocytes from MIRI rats in vitro

**Fig. 2:**
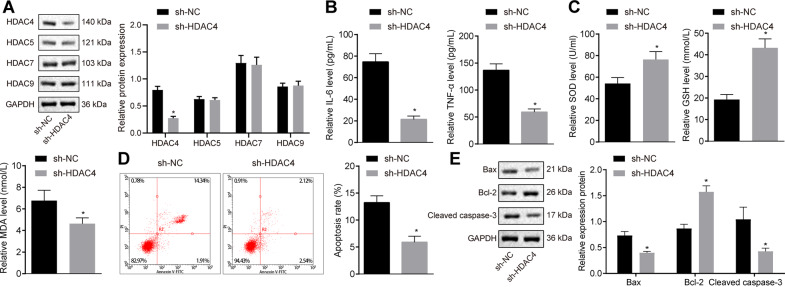
Silencing of HDAC4 protects cardiomyocytes from IRI in vitro. **A** HDAC4 silencing efficiency and the expression of HDAC5, HDAC7, and HDAC9 in cardiomyocytes evaluated by western blot analysis, normalized to GAPDH. **B** The levels of inflammatory factors (IL-6 and TNF-α) in cardiomyocytes determined by ELISA. **C** The levels of SOD, GSH, and MDA in the cardiomyocytes. **D** Apoptosis of cardiomyocytes detected by flow cytometry. **E** The expression of Bax, Bcl-2, and cleaved Caspase-3 protein in cardiomyocytes determined by western blot analysis, normalized to GAPDH. ^*^p < 0.05 vs. the cardiomyocytes transfected with sh-NC. Data were all measurement data and expressed as mean ± standard derivation. Comparisons between two groups were analyzed by unpaired *t-*test. The experiment was conducted three times independently.

In order to further verify the aforementioned in vivo findings, we isolated cardiomyocytes from rats with MIRI. HDAC4 was silenced in the isolated cardiomyocytes and its silencing efficiency was evaluated using western blot analysis, which revealed a substantially reduced expression of HDAC4 in cardiomyocytes transfected with sh-HDAC4 while the expression of its subtypes, HDAC5, HDAC7, and HDAC9, exhibited no changes (*p* < 0.05; Fig. 2A). Subsequently, we determined the levels of proinflammatory cytokines, and the contents of SOD, GHS, and MDA. In accordance with our in vivo results, HDAC4 silencing reduced the levels of IL-6 and TNF-α (*p* < 0.05; Fig. 2B), increased the content of SOD and GSH (*p* < 0.05; Fig. 2C), and reduced MDA content in cardiomyocytes, respectively (*p* < 0.05; Fig. 2C)./

Moreover, flow cytometry demonstrated that silencing HDAC4 could considerably diminish cell apoptosis (*p* < 0.05; Fig. 2D). Western blot analysis also illustrated a reduction in the expression levels of Bax and cleaved Caspase-3 and an increase in the expression levels of Bcl-2 in the absence of HDAC4 (*p* < 0.05; Fig. 2E). The aforementioned data supported the potential of HDAC4 silencing to mitigate myocardial injury in vitro.

### miR-206 is poorly expressed in the cardiomyocytes of rats with MIRI and its up-regulation of miR-206 could alleviate cardiomyocytes from MIRI rats in vitro

**Fig. 3:**
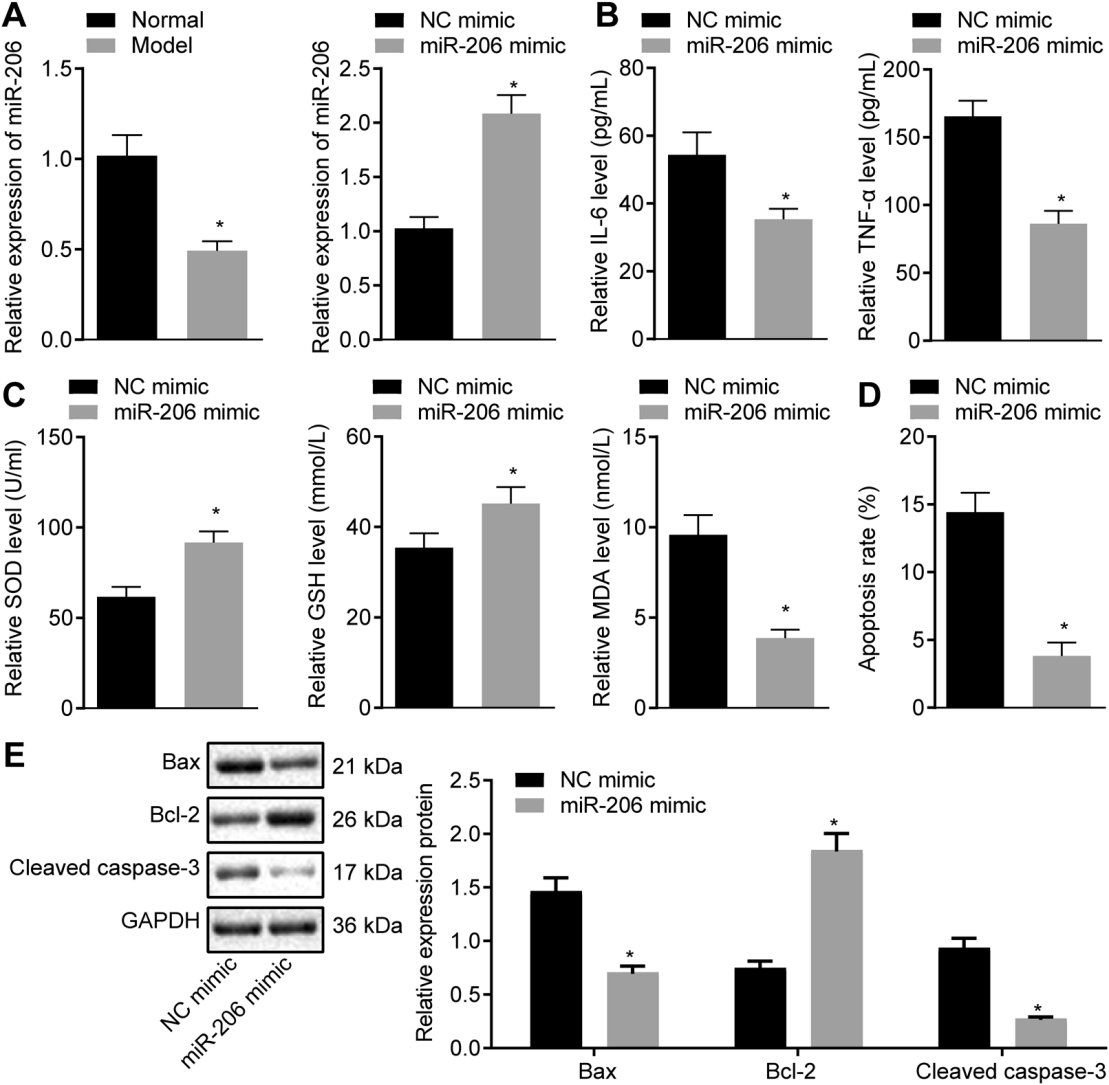
miR-206 is poorly expressed in the cardiomyocytes of rats with MIRI and its overexpression prevents cardiomyocytes from IRI in vitro. **A** Expression of miR-206 in cardiomyocytes determined by RT-qPCR, normalized to U6. **B** The levels of inflammatory factors (IL-6 and TNF-α) in cardiomyocytes detected by ELISA. **C** The levels of SOD, GSH, and MDA in cardiomyocytes. **D** Apoptosis of cardiomyocytes detected by flow cytometry. **E** Protein expression of Bax, cleaved Caspase-3, and Bcl-2 in cardiomyocytes determined by western blot analysis, normalized to GAPDH. ^*^p < 0.05 vs. the normal cardiomyocytes or the cardiomyocytes transfected with NC-mimic. Data were all measurement data and expressed as mean ± standard derivation. Comparisons between two groups were analyzed by unpaired *t-*test. The experiment was conducted three times independently.

The expression of miR-206 was measured in the cardiomyocytes of rats with MIRI by reverse transcription quantitative polymerase chain reaction (RT-qPCR), which demonstrated a significantly reduced miR-206 expression in IRI rats compared with the sham-operated rats (*p* < 0.05; Fig. 3A). In order to further define the role of miR-206 in the pathogenesis of MIRI, a gain-of-function study was performed in the cardiomyocytes. Subsequent findings revealed that miR-206 expression was evidently increased in miR-206 mimic-transfected cells as revealed by RT-qPCR (*p* < 0.05; Fig. 3A).

Subsequently, ELISA results displayed that miR-206 overexpression inhibited the levels of IL-6 and TNF-α (*p* < 0.05; Fig. 3B), elevated the contents of SOD and GSH (*p* < 0.05; Fig. 3C), and reduced the content of MDA in the cardiomyocytes (*p* < 0.05; Fig. 3C). Moreover, cells mimicking miR-206 expression were observably less susceptible to apoptosis compared with the cells expressing NC mimic (*p* < 0.05; Fig. 3D). In addition, up-regulated miR-206 also brought about a decreased Bax and cleaved Caspase-3 expression and up-regulated Bcl-2 expression in the cardiomyocytes (*p* < 0.05; Fig. 3E). Conjointly, miR-206 was poorly expressed in cardiomyocytes and overexpression of miR-206 could alleviate myocardial injury in vitro.

### HDAC4 silencing up-regulates miR-206 expression and inhibits activation of the MEKK1/JNK pathway in the cardiomyocytes of rats with MIRI

**Fig. 4:**
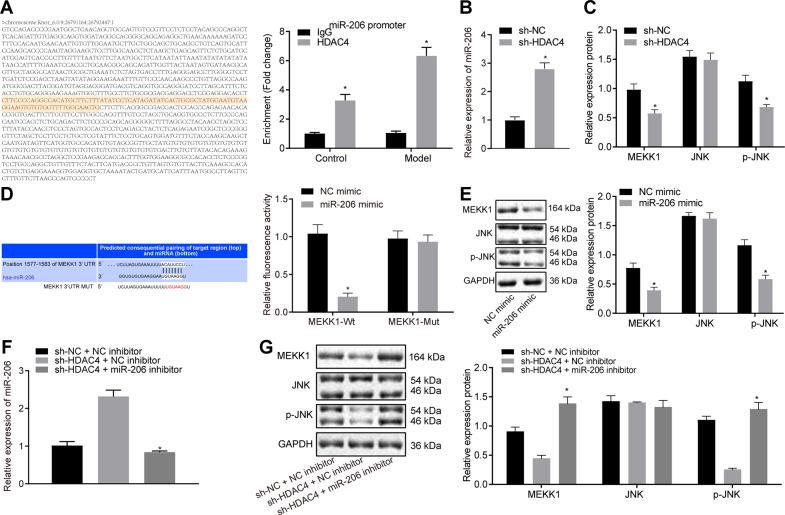
Silencing of HDAC4 elevates the expression of miR-206 and further inhibits the activation of the MEKK1/JNK pathway in cardiomyocytes from IRI rats. **A** The binding of HDAC4 to the promoter region of miR-206 identified by ChIP assay. **B** Expression of miR-206 in sh-HDAC4-treated cardiomyocytes determined by RT-qPCR. **C** Protein expression of MEKK1, JNK and p-JNK in sh-HDAC4-treated cardiomyocytes measured by western blot analysis, normalized to GAPDH. **D** The interaction between miR-206 and MEKK1 detected by dual luciferase reporter gene assay. **E** Protein expression of MEKK1 and JNK in miR-206 mimic-treated cardiomyocytes determined by western blot analysis, normalized to GAPDH. **F** Expression of miR-206 in cardiomyocytes transfected with sh-HDAC4 or sh-HDAC4 + miR-206 inhibitor measured by RT-qPCR. **G** The expression of MEKK1 and p-JNK in the cardiomyocytes transfected with sh-HDAC4 or sh-HDAC4 + miR-206 inhibitor measured by western blot analysis, normalized to GAPDH. ^*^p < 0.05 vs. the cardiomyocytes transfected with sh-NC, NC mimic, NC inhibitor, or sh-HDAC4 + NC inhibitor. Data were measurement data and expressed as mean ± standard derivation. Comparisons between two groups were analyzed by the unpaired *t-*test, and comparisons among multiple groups were analyzed by one-way ANOVA, followed by Tukey’s host hoc test. The experiment was conducted three times independently.

The results of chromatin immunoprecipitation (ChIP) assay in cardiomyocytes from sham-operated rats and rats with MIRI demonstrated that due to the high expression of HDAC4 in MIRI rats, significantly more HDAC4 was recruited to the promoter region of miR-206 in cardiomyocytes from rats with MIRI relative to cardiomyocytes from sham-operated rats (Fig. 4A). miR-206 expression detected by RT-qPCR showed an increase in cardiomyocytes transduced with sh-HDAC4 (*p* < 0.05; Fig. 4B). In addition, western blot analysis showed that the protein expression of MEKK1 and the extent of JNK phosphorylation were reduced in cardiomyocytes transfected with sh-HDAC4 (*p* < 0.05; Fig. 4C).

The interaction between miR-206 and MEKK1 was explored by means of dual luciferase reporter gene assay, which revealed a decrease in the luciferase activity in the cardiomyocytes co-transfected with miR-206 mimic and MEKK1 wild-type (wt) (*p* < 0.05), while no significant changes were observed in the luciferase activity of the cardiomyocytes co-transfected with miR-206 mimic and MEKK1-mutant (mut) (*p* *>* 0.05; Fig. 4D), indicating that miR-206 could target and negatively regulate MEKK1.

Moreover, western blot analysis showed a reduced protein expression of MEKK1 and extent of JNK phosphorylation in response to miR-206 mimic (*p* < 0.05; Fig. 4E). In addition, the miR-206 expression was down-regulated while the protein expression of MEKK1 and the extent of JNK phosphorylation were elevated in cardiomyocytes transfected with both sh-HDAC4 and miR-206 inhibitor (*p* < 0.05; Fig. 4F, G). The aforementioned data suggested that depletion of HDAC4 up-regulated the miR-206 expression and obstructed the activation of the MEKK1/JNK pathway in cardiomyocytes from MIRI rats.

### HDAC4 silencing restrains cell apoptosis via miR-206-mediated MEKK1/JNK disruption in cardiomyocytes from MIRI rats

**Fig. 5:**
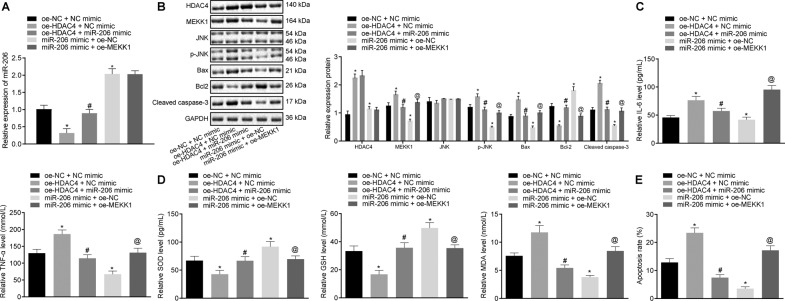
HDAC4 accelerates cell apoptosis by reducing miR-206 expression and activating the MEKK1/JNK axis in cardiomyocytes from IRI rats. Cardiomyocytes were transfected with oe-NC + NC mimic, oe-HDAC4 + NC mimic, oe-NC + miR-206 mimic, oe-HDAC4 + miR-206 mimic, miR-206 mimic + oe-NC, or miR-206 mimic + oe-MEKK1. **A** Expression of miR-206 in cardiomyocytes determined by RT-qPCR, normalized to U6. **B** Protein expression of HDAC4, MEKK1, JNK, p-JNK, Bax, cleaved Caspase-3, and Bcl-2 in cardiomyocytes determined by western blot analysis, normalized to GAPDH. **C** The levels of IL-6 and TNF-α in cardiomyocytes determined by ELISA. **D** Determination of SOD, GSH, and MDA contents in cardiomyocytes. **E** Apoptosis of cardiomyocytes detected by flow cytometry. Data were all measurement data and expressed as mean ± standard derivation. Comparisons among multiple groups were analyzed by one-way ANOVA, followed by Tukey’s host hoc test. ^*^p < 0.05 vs. the cardiomyocytes co-transfected with oe-NC and NC mimic; ^#^*p* < 0.05 vs. the cardiomyocytes co-transfected with oe-HDAC4 and NC; ^@^*p* < 0.05 vs. the cardiomyocytes co-transfected with miR-206 mimic and oe-NC. The experiment was conducted three times independently.

To investigate the function of HDAC4-dependent miR-206/MEKK1/JNK axis, we adopted RT-qPCR, which showed that up-regulation of HDAC4 brought about a down-regulated miR-206 expression in the cardiomyocytes and miR-206 mimic resulted in an elevated miR-206 expression, which was annulled by treatment with miR-206 mimic + overexpression (oe)-HDAC4. In the presence of miR-206 mimic, oe-MEKK1 did not impact miR-206 expression (Fig. 5A).

Western blot analysis revealed that in the presence of oe-HDAC4, miR-206 mimic did not affect HDAC4 expression, reduced MEKK1, Bax, and cleaved Caspase-3 expression and phosphorylation level of JNK, and elevated Bcl-2 expression in cardiomyocytes. However, oe-MEKK1 contributed to opposite trends in the presence of miR-206 mimic (Fig. 5B).

Moreover, miR-206 mimic exerted an inhibitory effect on the elevated IL-6 and TNF-α levels induced by HDAC4 overexpression (Fig. 5C). Furthermore, in cardiomyocytes overexpressing miR-206, further MEKK1 overexpression resulted in elevated levels of IL-6 and TNF-α (Fig. 5C).

In addition, in cardiomyocytes overexpressing HDAC4, miR-206 mimic augmented the contents of SOD and GSH, and diminished the content of MDA. In addition, in cardiomyocytes mimicking miR-206 expression, oe-MEKK1 triggered decreased contents of SOD and GSH and increased content of MDA (Fig. 5D). Furthermore, as shown in Fig. 5E, combination treatment with oe-HDAC4 and miR-206 mimic regressed the accelerated cell apoptosis induced by HDAC4 up-regulation, while combination treatment with miR-206 mimic and oe-MEKK1 increased cell apoptosis. These findings suggested that during IRI, up-regulated HDAC4 level to reduce miR-206 expression, which consequently activated the MEKK1/JNK pathway and ultimately rendered the cells to apoptosis in cardiomyocytes from MIRI rats.

## Discussion

MIRI is a severe cardiovascular condition, with highly intricate and complex pathogenic mechanism, which can be affected by multiple factors such as cytokines, chemokines, growth factors, free radical damages, and overload of calcium [24–26]. Existing research has revealed the potential cardioprotective benefits of HDAC inhibitors in MIRI [27, 28], thereby providing promising therapeutic approaches for this cardiovascular disease. The current study was set out to investigate the explicit function and molecular mechanism of HDAC4 in MIRI and the obtained results suggested that silencing of HDAC4 up-regulated the expression of miR-206, which inhibited the MEKK1/JNK pathway, thereby inhibiting the apoptosis of cardiomyocytes and alleviating MIRI.

Firstly, findings obtained in the current study revealed that HDAC4 was highly expressed in both myocardial tissues and cardiomyocytes following MIRI. Consistently, up-regulated HDAC4 level has been documented in oligodendrocyte progenitor cells in a study based on rat models with ischemic stroke [29, 30]. HDACs are also known to function as critical modulators for myocardial protection and cardiomyocyte survival [31]. Adding to this knowledge of HDACs, our study demonstrated that HDAC4 silencing facilitates the improvement of IRI-induced infarction and the resultant myocardial injury both in vivo and in vitro. In consistency with this, myocyte-specific overexpressing activated HDAC4 in mice stimulates MIRI substantiated by a reduction in ventricular functional recovery and increase in infarct size following IRI [32]. On the other hand, HDAC4 inhibition has been confirmed as an imperative stimulator for regeneration and cardiac function restoration [6]. Inhibition of HDAC4 can improve cardiac function and reduce myocardial infarction in mice suffering from ischemic-induced heart failure [33]. Additionally, HDAC4 deficiency can attenuate Ang II-induced cardiac hypertrophy in cardiomyocytes [34], as well as reducing cardio fibrosis in juvenile rats with overload-induced ventricular hypertrophy [35].

Furthermore, our findings highlighted that miR-206 was down-regulated in the cardiomyocytes of rats with MIRI, which is consistent with the findings obtained from other groups [15]. We further explored the function of miR-206 in MIRI and found that up-regulation of miR-206 confers a protective effect of up-regulated miR-206 against myocardial injury, evidenced by decreased levels of IL-6, TNF-α, and MDA yet increased content of SOD and GSH. miR-206 overexpression protects against cardiomyocyte apoptosis in vitro and in vivo in rodent models of myocardial infarction and IRI [36, 37]. IL-6 and TNF-α are fundamentally acknowledged as inflammatory indicators in rats suffering from cardiovascular disorders [38]. An existing study has documented compensated TNF-α and IL-6 release by miR-133b, thus alleviating myocardial injuries [39]. Moreover, another research identified the down-regulation of IL-6 and TNF-α as a sign of amelioration of heart disease [24]. Overexpression of miR-206 can undermine the aggravated hypoxia-induced MIRI by amplified long noncoding RNA RMRP [36]. MDA is one of the widespread biomarkers of oxidative stress and has undergone extensive investigation [40, 41]. SOD, as a ubiquitous enzyme, can principally protect tissues against oxidative distress via breakdown of superoxide radicals [42]. Also, GSH critically functions in antioxidant defense and serves as a vital regulator of pathways essential for maintaining body homeostasis [43]. More importantly, decreased levels of TNF-α, IL-6, and MDA have been observed along with increased SOD and GSH levels have been previously documented following alleviation of MIRI after oleuropein treatment [44]. Collectively, these findings supported the inhibitory role of miR-206 overexpression in MIRI.

An existing study reported that HDAC inhibition can bring about rapid alterations in miRNA levels [45]. HDAC4 has been documented to negatively regulate the expression of miR-200a [46]. Furthermore, HDAC4 is recruited to the miR-206 promoter in order to repress miR-206 transcription; HDAC4 stimulates miR-206’s target gene MRTF-A expression and facilitates fibrogenesis in hepatic stellate cells in a miR-206-dependent manner [47]. Although the inhibition of miR-206 ameliorates ischemia-reperfusion arrhythmia by targeting Cx43 [48, 49], we focused on the effect of HDAC4/miR-206 axis on IRI in the current study, which has not been previously reported. In the current study, we uncovered a similar targeting relationship between miR-206 and MEKK1. Interestingly, MEKK1 and JNK are radically repressed by miR-206, thus implicating their function in skeletal muscle development [16]. miR-206 regulates cell movements during zebrafish gastrulation by regulating the JNK pathway [47]. Meanwhile, the MEKK1/JNK pathway has been suggested as a contributor of IRI and myocyte apoptosis [50]. In addition, both miR-140-5p overexpression and HDAC4 silencing have been revealed to reduce the apoptosis of cardiomyocytes, thus exerting cardioprotective effects against diabetic cardiomyopathy [51]. Inhibition of HDAC4 attenuates neuronal apoptosis via reduction of JNK/c-Jun activity during early brain injury following subarachnoid hemorrhage [52]. Together, our findings in conjunction with existing evidence indicate that HDAC4 down-regulation can augment miR-206 expression and subsequently inactivate the MEKK1/JNK pathway, leading to repression of cardiomyocyte apoptosis and further attenuation of IRI.

**Fig. 6:**
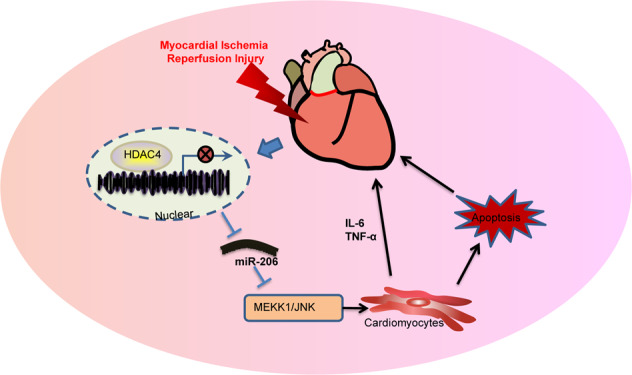
The mechanistic diagram. HDAC4 down-regulates the expression of miR-206 to promote cardiomyocyte apoptosis and oxidative stress, thereby inducing MIRI by activating MEKK1/JNK pathway.

In conclusion, findings obtained in the current study demonstrated that HDAC4 silencing could up-regulate the expression of miR-206 and inhibit the activation of the MEKK1/JNK pathway, thereby reducing cardiomyocyte apoptosis and inhibiting oxidative stress, and exerting a protective effect on MIRI (Fig. 6). Our study validates the cardioprotective effect of miR-206 up-regulation, which may be a promising viable target for MIRI treatment. However, since siRNA silencing of HDAC4 was only ~50% in relation to the non-targeting controls, analysis of other HDAC isoforms with superior silencing efficiency should be conducted in the future. Also, further studies are warranted to determine whether the HDAC4/miR-206/MEKK1/JNK axis is involved in other types of cell death and to confirm the clinical application of the corresponding axis in treating MIRI.

## Materials and methods

### Ethics statement

The current study was conducted with approval of the animal ethics committee of the Zhengzhou University People’s Hospital, Henan Provincial People’s Hospital,Central China Fuwai Hospital (protocol No. 20201230c0400510[376]) and performed in accordance with the recommendations in the Guide for the Care and Use of Laboratory Animals published by the US National Institutes of Health. Extensive measures were undertaken to minimize the number and suffering of the included animals.

### Establishment of rat models of IRI

A total of 105 specific pathogen-free male Sprague-Dawley (SD) rats (age: 8–10 weeks; weight: 310–400 g) were included in the current study, among which 45 rats were subjected to sham operation and the remaining 60 were subjected to establishment of MIRI models. The heart function of all rats was evaluated using small animal ultrasound (Vevo). The establishment of the IRI model was conducted as the previously described method [53]. Briefly, rats were intraperitoneally anesthetized using intraperitoneal injections with 20% urethane and fixated in a supine position, followed by measurement of their body temperature, blood pressure, heart rate, and average arterial pressure. Next, mechanical ventilation was performed after tracheal intubation. An incision was then made at about 0.5 cm from the left edge of the sternum between the third and fourth intercostal space of the left side of the ribcage. After dissection and retraction of the pectoral muscle, the 3rd intercostal space was exposed. Subsequently, thoracotomy was then performed to expose the heart. Afterwards, left anterior descending (LAD) coronary artery was identified and ligated using a 5-0 silk suture. Pallor appearance of the myocardium followed abruptly by cyanosis and elevated ST segment on the electrocardiograph was regarded as an indicator of myocardial ischemia establishment. The LAD coronary artery of sham-operated rats was penetrated using 50 silk threads without ligation of the suture. The tissues were harvested immediately after 24 h of reperfusion. After 30 min of ligation, the suture was re-opened for 2 h. The reperfusion model was considered to be successfully established when the ST segment descended or the QRS wave progressively reverted to normal levels. Nine rat casualties were encountered during the modeling process, with failure in three rats. The success rate of modeling was calculated to be 76.67%, and 45 were chosen for subsequent experimentation, with 5 rats in each group used for TTC staining. Heart tissues of rats in each group were extracted and sliced into 8 transverse sections (2 mm each), and sections were then stained with 0.75% TTC solution. The infarct size was determined by experienced blind researchers using the SigmaScan Pro5 software for planimetric measurement. Planimetric-determined infarct size of each section was normalized to section weight and the mean value of the sum of section weight was obtained.

### Isolation of primary cardiomyocytes

Normal rats and MIRI modeled rats were intraperitoneally injected with heparin (5000 U/kg). After 15–20 min, the rats were intraperitoneally anesthetized with 1% pentobarbital sodium. Next, the heart along with the aortic arch was isolated and immersed in ice-cold (4 °C) calcium-free solution. Subsequently, the heart along with the aortic arch was perfused with the Langendorff system as per a previously reported method [54]. After the heart became soft, the aortic arch was removed and the heart was transferred to collagenase type 1 solution (SCR103, Sigma-Aldrich, St Louis, MO, USA). The ventricular tissues were dissected into small sections (1 mm × 1 mm × 1 mm) and resuspended using a pipette with the blunt end tip. The tissues were then filtered using a cell strainer (75 μm mesh) and maintained at room temperature for 10–15 min. The precipitates, which were considered as cardiomyocytes, were resuspended at room temperature for 10 min. The aforementioned protocols were repeated three times to isolate the enriched cardiomyocytes. Afterwards, the cells were plated in culture dishes pre-coated with the mouse laminin at a density of 0.5–1 × 10^4^ cells/cm^2^.

### Cell treatment and in vivo injection

The plasmids of miR-206 mimic, miR-206 inhibitor, overexpression (oe)-MEKK1, and oe-HDAC4 were provided by Shanghai Genechem Co., Ltd. (Shanghai, China). The lentivirus harboring shRNA against HDAC4 (sh-HDAC4) was constructed by Suzhou GenePharma Co., Ltd. (Suzhou, Jiangsu, China). The cardiomyocytes of MIRI rats or normal rats were transferred to a 6-well plate for 24 h and then transfected with the corresponding plasmids. Next, 250 μL Dulbecco’s modified Eagle’s medium (DMEM; Gibco, Carlsbad, CA, USA) was used to dilute 8 μL of the HDAC4 interference sequence and 6 μL of Lipofectamine 2000, which was then mixed, allowed to stand for 20 min, and added to the culture well. After gentle fusing, the culture was continued in a 5% CO_2_ incubator at 37 °C, and the medium was renewed with DMEM containing 10% fetal bovine serum (FBS) and penicillin/streptomycin after 8 h. The cells were collected 36 h after transfection. For in vivo injection, the recombinant lentivirus specifically targeting cardiomyocytes harboring sh-HDAC4 or sh-NC (10^8^ pfu/mL/rat with normal saline as solvent) was intramyocardially injected into the rats. The tissues were obtained immediately after 24 h of reperfusion.

### H&E staining

Heart tissues were dissected into small sections and fixed with 10% neutral formalin. Next, the fixed tissues were paraffin-embedded and then sectioned. The tissue sections were subjected to H&E staining for histological analysis as previously described [55]. Each section underwent observation under an optical microscope (XP-330, Shanghai Bingyu Optical Instrument Co., Ltd., Shanghai, China) in a double-blind-manner. Three random visual fields were selected to evaluate myocardial congestion, hemorrhage, fibrosis, necrosis, and degeneration. The scoring criteria were as follows: 0 indicated no lesion; 0–1 indicated lesions were less than 1/4 of the designated area; 1–2 indicated lesions ranged from approximately 1/4−1/2 of the designated area; 2–3 indicated lesions ranged from approximately 1/2−3/4 of the designated area; and 3–4 indicated lesions were greater than 3/4 of the designated area.

### TTC staining

The 5 mm myocardial sections from heart samples of 5 rats in each group were placed in 1% TTC solution (AMRESCO, USA) and incubated at 37 °C under dark conditions for 10 min. Afterwards, the sections were fixed with 10% formalin for 2 h and observed under a stereoscopic microscope (Zeiss, Germany). The white part was indicative of infarct area, while red part was indicative of non-infarct area. The Image-Pro Plus 6.0 software (Media Cybernetics) was adopted to calculate the infarct area (%) = infarct area/transverse section area × 100%.

### TUNEL assay

Frozen sections were fixed with 4% paraformaldehyde for 1 h, blocked with blocking solution for 10 min, and dripped with the penetrating solution on ice for 5 min. Cell apoptosis was determined using a TUNEL Apoptosis Kit (Roche, Basel, Switzerland). The nucleus was stained with 4ʹ,6-diamidino-2-phenylindole (Sigma-Aldrich). Immunofluorescence was performed under a fluorescence microscope (Carl Zeiss, Jena, Germany). Apoptotic nuclei and total nuclei were calculated at ×200 magnification.

### ELISA

Blood samples were extracted from the orbital sinus was centrifuged at 3500*g* and the serum was collected and stored at −80 °C. Serum levels of IL-6 and TNF-αwere measured using the murine IL-6 and TNF-α ELISA kits (MSKbio Co., Ltd., Wuhan, Hubei, China) in strict accordance with the manufacturer’s instructions.

After 24 h of culture, the cell medium was collected and centrifuged at 1000*g* at room temperature for 10 min, followed by supernatant collection. Subsequently, ELISA was performed in compliance with the provided instructions (MSKbio Co., Ltd., Wuhan, Hubei, China) to determine the levels of TNF-α (69-22452) and IL-6 (69-30490).

### Determination of SOD activity, reduced GSH and MDA

Myocardial tissues (125 mm^3^) from MIRI rat or normal rats were collected from each experimental group and centrifuged after the addition of 1 mL PBS at 10,000*g* and 4 °C for 10 min, after which the supernatant was harvested. The protein concentration was measured using a bicinchoninic acid kit (P0011, Beyotime, Shanghai, China). The contents of MDA, SOD and GSH in the myocardial tissues were determined using MDA (A003-1-2), SOD (A001-3-2), and GSH (A006-2-1) assay kits (Nanjing JianCheng Bioengineering Institute, Nanjing, China), respectively.

### Dual luciferase reporter gene assay

Reporter plasmids containing wt MEKK1 or mut MEKK1 (NM_005921.2) were provided by Shanghai GenePharma Co., Ltd. (Shanghai, China). Next, two plasmids were co-transfected with the NC mimic and miR-206 mimic into 293T cells, respectively. After 48 h of culture, the cells were harvested. The luciferase activity was subsequently measured in accordance with the protocols provided with the GenecoPoeia’s Dual Luciferase Assay Kit (D0010, Beijing Solarbio Science & Technology Co., Ltd., Beijing, China). The fluorescence intensity was then measured using a Promega’s Glomax 20/20 Luminometer (E5311, Zhongmei Biotechnology Co., Ltd., Shaanxi, China). Luminescent signal reflecting activation of the target reporter gene was compared based on the ratio of the firefly relative light luciferase unit (RLU) to the Renilla RLU.

### ChIP assay

The cardiomyocytes transduced with sh-NC and sh-HDAC4 were collected. When the cell density reaches 1 × 10^6^ cells/10 cm culture dish, the original culture medium was discarded, incubated with 1% formaldehyde at 37 °C for 10 min, and added with 2.5 mM glycine on ice for 5 min to stop cross-linking, followed by digestion and centrifugation to obtain the cell pellet. The cells were resuspended in 200 μL sodium dodecyl sulfate (SDS) lysis buffer, placed on ice for 10 min for cross-linking reaction, and the chromatin DNA was fragmented with ultrasound. The cells were centrifuged at 14,000 rpm and 4 °C for 10 min, after which the supernatant was attained. After being diluted with ChIP dilution buffer containing protease inhibitors, the supernatant was incubated with blocking solution at 4 °C for 30 min. After centrifugation at 1000 rpm and 4 °C for 1 min, a small amount of supernatant served as Input, and the remaining supernatant was added with HDAC4 antibody (ab12171, 1:1000, anti-rabbit, Abcam, Cambridge, UK) or NC Immunoglobulin G (IgG) (ab172730, 1:1000, anti-rabbit, Abcam), followed by incubation overnight at 4 °C. The supernatant was incubated with cross-linked agar at 4 °C for 1 h to collect antibody/transcription factor complexes, followed by centrifugation at 1000 rpm and 4 °C for 1 min. After discarding the supernatant, the complex was eluted. The eluted supernatant and Input DNA was added with 20 μL of 5 mol/L NaCl and water-bathed at 65 °C for 4 h for decrosslinking. The DNA was purified and recovered after Proteinase K digestion for protein removal. With the recovered DNA as a template, RT-qPCR was performed to detect the expression of DNA binding to the miR-206 promoter. The specific primers for ChIP assay of miR-206 promoter: F: 5ʹ-CTACTTATGCAGCTAGAGATACAAG-3ʹ and R: 5ʹ-ACTTCCAATAAGTCTTTGACCCATG-3ʹ.

### RT-qPCR

Total RNA content was extracted using the TRIzol reagent. The PolyA tailing detection kit (B532451, Sangon, Shanghai, China; containing universal PCR primer R) was applied to obtain the complementary DNA (cDNA) of the miRNA with the PolyA tail in strict accordance with the provided instructions. The primer for miR-206 was designed and synthesized by Takara (Tokyo, Japan) (Table S1). RT-qPCR was performed in triplicate using the SYBR^®^ Premix Ex Taq^TM^ II Kit (RR820A, Takara, Tokyo, Japan) using the ABI 7500 instrument (Applied Biosystems, Foster City, CA, USA). The transcriptional levels of miR-206 were estimated based on relative quantification (2^-△△CT^ method), and then normalized to U6 mRNA.

### Western blot

Total protein was extracted from cells using the radioimmunoprecipitation assay (RIPA) lysis solution (R0010, Beijing Solarbio Science & Technology Co., Ltd., Beijing, China) supplemented with phenylmethylsulfonyl fluoride (PMSF) and phosphatase inhibitor. Then the extracted protein was separated by conducting 10% SDS-polyacrylamide gel electrophoresis and was transferred onto polyvinylidene fluoride membranes. A membrane blockade was subsequently conducted using 5% skim milk powder for 1 h at room temperature, and then probed with the diluted primary rabbit antibodies against HDAC4 (1:1000, ab12172), MEKK1 (1:1000, ab138662), JNK (1:1000, ab179461), p-JNK (1:1000, ab124956), Bax (1:5000, ab32503), Bcl-2 (1:2000, ab59348), cleaved Caspase-3 (1: 500, ab49822), and glyceraldehyde-3-phosphate dehydrogenase (GAPDH; 1:10,000, ab181602) overnight at 4 °C. The following day, the membrane was re-probed with horseradish peroxidase-conjugated secondary antibody goat anti-rabbit IgG (1:5000, ab205718) for 1 h. The aforementioned antibodies were provided by Abcam. The membrane was then developed using an enhanced chemiluminescence (ECL) reagent (ECL808-25, Biomiga, USA) for 1 min at room temperature and exposed to X-ray for imaging (36209ES01, Qianchen Biotechnology Co., Ltd., Shanghai, China). GAPDH served as an internal reference, and the ratio of the gray value of the target band to that of the internal reference band was regarded as the relative protein expression.

### Apoptosis assay by flow cytometry

Cells were treated with 0.25% trypsin after 36 h of transduction. Afterwards, the cells were collected, rinsed twice with PBS, and resuspended in 200 μL of the binding buffer. Next, the cells were supplemented with 10 μL of Annexin V-fluorescein isothiocyanate (FITC; ab14085, Abcam) and 5 μL of propidium iodide (PI), which was then gently infused and reacted under dark conditions at room temperature for 15 min. Next, 300 μL of the binding buffer was added to the mixture. Cell apoptosis was subsequently evaluated using a flow cytometer (BD FACSCanto II, Image Trading Co., Ltd., Beijing, China) at an excitation wavelength of 488 nm.

### Statistical analysis

Statistical analyses were performed using the SPSS 21.0 statistical software (IBM Corp, Armonk, NY, USA) was adopted for statistical analyses. Measurement data were expressed as mean ± standard deviation. Data conforming normal distribution and homogeneity of variance between two groups were compared using the unpaired *t-*test while data comparisons among multiple groups were performed using one-way analysis of variance (ANOVA) with the Tukey’s post hoc test. A value of *p* < 0.05 was considered to be statistically significant.

## Supplementary information

Table S1.

Author-contribution-form.

## Data Availability

The datasets generated and/or analyzed during the current study are available from the corresponding authors on reasonable request.
