# Supplementary material for: Histone deacetylase HDAC4 participates in the pathological process of myocardial ischemia-reperfusion injury via MEKK1/JNK pathway by binding to miR-206
Source: Cell Death Discov. 2021 Sep 15;7:240. doi: 10.1038/s41420-021-00601-1 (PMC8443671; doi:10.1038/s41420-021-00601-1)
Supplement: Supplementary file 1 — Table S1. [file 41420_2021_601_MOESM1_ESM.docx]

**Table S1** The primer sequences for RT-qPCR

| Target | Primer sequences (5’ - 3’) |
| --- | --- |
| miR-206 | F: 5’-TGGAATGTAAGGAAGTGTGTGG-3’ |
| U6 | F: 5’-GCTTCGGCAGCACATATACTAAAAT-3’ |

Note: miR-206, microRNA-206; RT-qPCR, reverse transcription-quantitative polymerase chain reaction; F, forward; R, reverse.
