# Supplementary material for: Histone deacetylase HDAC4 participates in the pathological process of myocardial ischemia-reperfusion injury via MEKK1/JNK pathway by binding to miR-206
Source: Cell Death Discov. 2021 Sep 15;7:240. doi: 10.1038/s41420-021-00601-1 (PMC8443671; doi:10.1038/s41420-021-00601-1)
Supplement: Supplementary file 2 — Author-contribution-form. [file 41420_2021_601_MOESM2_ESM.pdf]

DECLARATION OF CONTRIBUTIONS TO ARTICLE

ADMC

Manuscript Number: CDDISCOVERY-21-1612R

Journal Name: *Cell Death Discovery* (the 'Journal')

Proposed Title of the Contribution:

Histone deacetylase HDAC4 participates in the pathological process of myocardial ischemia-reperfusion injury via MEKK1/JNK pathway by binding to miR-206 (the 'Contribution)

Author(s):

Qingman Li, Lijie Zhu, Fangqing Niu, Qingmin Li, Che Wang, Honghui Yang, Chuanyu Gao (the 'Authors')

For all *CDDis*covery articles, each person named as an author in the published version must be able to show he or she has contributed substantially to the article.

Authorship credit should be based on 1) substantial contributions to conception and design, acquisition of data, or analysis and interpretation of data; 2) drafting the article or revising it critically for important intellectual content; and 3) final approval of the version to be published. Authors should meet conditions 1, 2 and 3.

Any person who cannot be shown to have made a substantial contribution to the article cannot be listed as an author in the final version. The name of any person who is deemed to have made a minor contribution can, however, appear in the Acknowledgments section of the article.

Please complete the table below to indicate the contributions of all named authors to the manuscript.

Author Full Name:	Specification of Contribution to the Manuscript:
Qingman Li	Conceptualization (Lead), Data curation (Equal), Investigation (Lead), Methodology (Lead), Writing-original draft (Lead), Writing-review & editing (Equal)
Lijie Zhu	Data curation (Equal), Investigation (Supporting), Methodology (Supporting), Validation (Equal), Writing-original draft (Supporting), Writing-review & editing (Equal)
Fangqing Niu	Data curation (Equal), Formal analysis (Equal), Software (Equal), Validation (Equal), Writing-review & editing (Equal)
Qingmin Li	Formal analysis (Equal), Software (Equal), Visualization (Equal), Writing-review & editing (Equal)
Che Wang	Resources (Equal), Validation (Equal), Visualization (Equal), Writing-review & editing (Equal)
Honghui Yang	Formal analysis (Equal), Resources (Equal), Supervision (Equal), Visualization (Equal), Writing-review & editing (Equal)
Chuanyu Gao	Formal analysis (Equal), Resources (Equal), Software (Equal), Supervision (Equal), Writing-review & editing (Equal)

Please complete the table below to indicate the contributions of all named authors to the figures.

Figure 1:

Qingmin Li, Che Wang, Honghui Yang

Figure 2:

Qingmin Li, Che Wang, Honghui Yang

Figure 3:

Qingmin Li, Che Wang, Honghui Yang

Figure 4:

Qingmin Li, Che Wang, Honghui Yang

Figure 5:

Qingmin Li, Che Wang, Honghui Yang

Figure 6:

Qingmin Li, Che Wang, Honghui Yang

Signed for and on behalf of the Author(s):	Print Name:	Date:
Honghui Yang Chuanyu Gao	Honghui Yang & Chuanyu Gao	2021/6/29
